# Predictors of Positive and Negative Emotions Experienced by Poles during the Second Wave of the COVID-19 Pandemic

**DOI:** 10.3390/ijerph182211993

**Published:** 2021-11-15

**Authors:** Estera Twardowska-Staszek, Irmina Rostek, Krzysztof Biel, Anna Seredyńska

**Affiliations:** Institute of Educational Sciences, Jesuit University Ignatianum in Krakow, 31-501 Cracow, Poland; irmina.rostek@ignatianum.edu.pl (I.R.); krzysztof.biel@ignatianum.edu.pl (K.B.); anna.seredynska@ignatianum.edu.pl (A.S.)

**Keywords:** pandemic, COVID-19, mental health, emotions, optimism, satisfaction with life, coping with stress

## Abstract

The objective of the research was to specify the predictors of positive and negative emotions experienced by Poles during the second wave of the COVID-19 pandemic. The researchers used the following standardized measurement tools: emotions (PANAS), mood (UMACL), satisfaction with life (SWLS), optimism (LOT-R), and coping with stress (CISS). They also used a questionnaire to collect sociodemographic information and data concerning COVID-19 infections. In total, 595 participants (80.50% women) aged 18–75 participated in the research. It was concluded that the predictors of positive emotions included a task-oriented coping style, level of satisfaction with life, being a man, hedonic tone in the description of mood, and being an employed student. The negative predictors of positive emotions included emotion-oriented coping and the level of energetic arousal in the description of mood. The predictors of negative emotions were tense arousal in the description of mood, emotion-oriented coping, being over 60 years of age, and changes in respondents’ standard of living. The negative predictors of negative emotions included living in a medium-sized town or in a village. The research conclusions encourage us to pay special attention to possible at-risk groups threatened with mental health disorders and to factors that protect people against negative psychological consequences of the COVID-19 pandemic.

## 1. Introduction

The SARS-CoV-2 coronavirus, which was first diagnosed in Wuhan, China, in December 2019, was first recognized in a Polish patient on 4 March 2020 [1]. This day is the beginning of the first wave of the epidemic in Poland. Over the following 2 months, the government implemented various preventive measures. First, mass events were cancelled, followed by severe restrictions on international travel to and from Poland. Within the following few weeks, educational institutions at all levels were closed, and then switched from teaching and learning on-site to teaching and learning online. Serious limitations in movement were introduced (parks, beaches, boulevards, and, finally, forests were closed), along with fines for breaches of those restrictions. In addition, the obligation to cover one’s mouth and nose in public spaces was implemented [1].

The first analyses concerning the psychological consequences of the epidemic were performed in China as early as the beginning of 2020 [2]. The first reviews of research results [3], which were published in April, summed up the information from Chinese observations and articles describing the possible influence of the pandemic on mental health. In additiom, publications prepared in many other countries (e.g., Brazil, Canada, Iran, Iraq) suggested that the epidemic may decrease people’s level of functioning in a subjective dimension, e.g., by increasing one’s sense of insecurity, loneliness, anxiety, and stress, and in an objective dimension, e.g., through a worsening in the economic situation of both individuals and whole countries. In addition, the first pandemic reports attempted to indicate groups at risk of not coping well with the situation. Such groups included people infected with COVID-19, their families, people who had pre-existing conditions before the pandemic, and health service employees [3].

The fear of becoming infected with the virus affected people all over the world. A Gallup poll conducted in March in 19 countries indicated that more than half of respondents were afraid of becoming infected or worried about their family members who may become infected with the coronavirus. The most apprehensive respondents were the Italians, among which 90% expressed anxiety, while the least worried were the Japanese and the American respondents—52% of respondents in those countries were fearful of the coronavirus infection. Interestingly, the relatively lowest levels of anxiety among the Japanese and Americans had nothing to do with their evaluation of both the current and future situation in their countries. Only 23% of the Japanese and 42% of the American respondents believed that their governments had coped with the pandemic very well. In addition, when asked about the predicted end of the pandemic, the citizens of those countries expressed poor optimism: only 11% of Japanese and 28% of Americans believed that life would return to normal by the end of 2020 [4].

The first analyses concerning the mental state of Poles during the pandemic were performed in March (e.g., [5,6,7,8] and April 2020 (e.g., [9,10,11,12]). The research conducted in March [4] showed that, at first, Poles’ emotional reactions were not dominated by negative emotions. The people researched who kept personal diaries experienced happiness and relaxation twice as often as anger, anxiety, or sadness. Later, a repeated cross-sectional survey was conducted among students of Polish universities in March and April (using the Depression Anxiety Stress Scales, DASS) [6], which revealed that depression indices increased in a significant manner, while anxiety and stress indices increased in a statistically insignificant manner. In other studies among students [7], which were conducted in March and April (with the use of the Generalized Anxiety Disorder scale, GAD-7), researchers found that 65% of students experienced fear, while 14% reported a severe anxiety disorder. In addition, 56% of the students who participated in the survey experienced a high or very high level of stress (measured with the Perceived Stress Scale, PSS-10.

The research conducted in March on a representative sample of Poles [8] indicated a high level of nervousness in the general population (in the self-evaluation of nervousness due to the pandemic on a scale from 1 to 100, M = 63.44). Most stress factors were related to other people: strangers as potential and irresponsible virus spreaders (75% of the people researched identified with this fear), as well as family members as possible victims of the virus (72%). People’s fear of contracting the virus was less intensive (59%). In addition, the level of stress was measured during this study (with the use of a tool prepared on the basis of GAD-7 and Patient Health Questionnaire, PHQ-9) (in the evaluation of the scale from 1 to 5, M = 2.76). In April, the level of nervousness decreased by a small, but statistically significant degree (M = 60.20), while people’s fears mainly related to the financial crisis (80%) and to the inefficiency of the health care system (79%). The fear of becoming infected with the virus was still lower than fear about the health of family members. The level of stress decreased slightly, but in a statistically significant manner (M = 2.70).

The research conducted among the general population of Poland in April [9] indicated that 77% people were afraid of becoming infected, and 71% reported anxiety at different levels of intensity (44% of the results might suggest the occurrence of general anxiety disorder). Retrospectively, the people surveyed (85%) indicated feeling nervousness, anxiety, and tension within 14 days preceding the survey (utilizing the GAD-7). Other studies of a similar nature [10] showed a similar picture: 52.82% of the people surveyed using the GHQ–28 (General Health Questionnaire-28) obtained a sten score of 7 or more, while 26.18% obtained sten scores at level 9 or 10 (which suggests the occurrence of serious mental health problems). The results concerning stress were similar (research was conducted utilizing the PSS-10): 53% of those surveyed obtained a sten score of 7 or higher (which confirms the high level of stress they experienced). The results obtained using the same scale (PSS-10) in other studies [11] indicated a moderate level of stress in 57% of people, while a high level of stress was found among 29% of respondents.

The first weeks of the pandemic were characterized by a large fluctuation of emotions: in the abovementioned studies conducted by Gallup [4], which were conducted between the end of March and the beginning of April. In 9 out of 13 countries, the number of people afraid of contracting the coronavirus increased, while between the beginning of April and the beginning of June, in 9 out of 13 countries, the number of such people decreased.

In the last week of April, the process of removing restrictions began in Poland, which was primarily motivated by economic factors. Despite the increase in the number of infections, the Polish prime minister, Mateusz Morawiecki, announced that the pandemic was “in retreat”, which was reflected in the emotions experienced by Poles. At the turn of May and June, the level of nervousness due to the epidemic dropped (M = 52.8). Furthermore, specific symptoms of such nervousness changed, the most intensive of them being uncertainty related to a possible economic crisis (63%). In total, 45% of Poles were afraid of contracting COVID-19, while 60% were afraid that their family members would fall ill [8]. Compared to May, in July the intensity of depression and general anxiety disorder symptoms decreased (analyzed using the GAD-7 and PHQ-9) [13].

Inasmuch as in the first phase of the pandemic. the occurrence of three basic approaches to the situation among those surveyed could be noticed [14]: the involved approach, which constituted almost half of people in the researched group; and the cautious and indifferent approaches, which each constituted a quarter of respondents. The improvement in the emotional state of Poles in the summer was accompanied by a kind of denial of the pandemic problem. Such denial was confirmed by the results of a large survey conducted by Ipsos [15] in different countries, the results of which were published in September. In the question concerning the willingness to be vaccinated against COVID-19, Poland (in the group of 27 countries) placed second to last (just before Russia), with only 54% people prepared to be vaccinated. Moreover, while 45% of respondents from the 27 countries surveyed in September (also by Ipsos) [16] declared that, at that moment, the biggest problem in their countries was COVID-19, in Poland, the percentage was only 38%. The most serious concerns (after the pandemic) of the world’s population included unemployment, poverty, inequalities, crime, and violence, but Poles were not afraid of these. Polish people were not worried about losing a job (the second to last position, with 19%; 39% being the mean for all the analyzed countries); they were the least worried about poverty and social inequalities (17%, with 30% being the average result for all the countries); and they were not afraid of crime and violence (6% compared to a 27% average for the other countries). The Poles’ most serious problems (which were largely influenced by the political and economic situation in the country) included those related to the functioning of the health service (45%—the second position among all the countries, the mean being 21%) and corruption (35%—the eighth position, with a mean of 27%). In addition, Poles’ worries that were greater than the world’s mean were connected with financial assistance provided by the state, e.g., measures related to taxation or inflation.

Along with the second wave of the pandemic in October, due to a high increase in the number of infections, educational, cultural, sport institutions, and restaurants were again closed. Following an increase in infections in November amounting to around 20 thousand cases per day and a record-breaking number of deaths (more than 600 people a day), in the middle of December, a national lockdown was introduced. This resulted in the closure of hotels, shopping centers, ski resorts, limitations in the number of people meeting in family houses during Christmas, as well as a ban on movement from one place to another on New Year’s Eve. Moreover, at the end of October, women’s protests against a toughening of the abortion laws began. In the second half of December, the number of infections decreased, and the first COVID-19 vaccination was administered.

Longitudinal studies revealed that, at the end of the year, people’s nervousness related to the pandemic had returned to levels observed the previous April, but the main object of their worries changed (69% of the surveyed people were mainly worried about limited access to health services, 61% were worried about the country’s financial situation) [8]. Comparing to July, the number of people from the high-risk group of patients with a clinically important intensification of depression symptoms (29% for women and 24% for men) and anxiety symptoms (31% for women and 26% for men) increased significantly [13]. In cross-sectional studies, the average level of anxiety (analyzed using the Hospital Anxiety and Depression Scale, HADS) had increased, with anxiety disorders occurring among 32.69% of the people surveyed. Similar to the longitudinal studies, 23.14% of respondents revealed depression symptoms, and the average level of stress (measured with PSS) was high [17]. In the case of 59.2% participants, the mean result of the GHQ-28 indicated the occurrence of minor mental disorders [18].

The Ipsos research that was conducted at that time showed that, in November, the primary concern of Poles was the coronavirus (55% of respondents indicated the pandemic as one of the three most important problems in the country). In addition, compared to September, the level of anxiety related to the functioning of the health service increased (53%) [19]. In December, the level of people’s anxiety about the coronavirus decreased (to 42%), while the condition of the Polish health service was, again, the main concern of respondents (53%). When asked whether they believe that the situation in the country was moving in the right direction, 82% of Poles declared “no.” This was the highest percentage in all the 27 countries surveyed [20].

Three conclusions can be drawn from the above results. First, due to a high dynamic of change, it is necessary to conduct further research (both cross-sectional and longitudinal) in the following weeks and months of the pandemic. Second, because of significant cultural differences in experiencing pandemic stress, it is necessary to consider the elements that go beyond the virus threat in diagnosing the mental condition of Poles. Third, it is necessary to carefully analyze people’s mental health and well-being, considering not only the most common aspects such as anxiety, stress, and depression, which were noticeable at the very beginning of the pandemic, but also more subtle issues related to a person’s emotional functioning.

The research described in this article constitutes the first stage of a broader research project which, in its assumptions, aims to look for predictors of emotional wellbeing in the context of such variables as sociodemographic data, satisfaction with life, optimism, and styles of coping with stress. According to pre-pandemic surveys, experiencing positive emotions was related to good mental health and social adjustment, as well as rare episodes of anxiety, while experiencing negative emotions may be connected with decreased psychosocial functioning [21]. Thus, the objective of our research was to learn about the predictors of the positive and negative emotions of adult Poles during the second wave of the COVID-19 pandemic. It is assumed that the research results will be the basis for introducing psychological interventions, the aims of which are to prevent and reduce negative consequences for people’s mental health.

## 2. Materials and Methods

### 2.1. Participants

Due to the epidemiological situation, the research was conducted online with participants who were asked to complete an online survey shared through personal contacts (text messages and e-mail) and on social media (Facebook). To be included in the survey, participants had to be over 18 years of age and a resident of Poland.

### 2.2. Measures

Sociodemographic variables and data related to COVID-19 infection were collected using an ad hoc self-made questionnaire. The questionnaire comprised 7 sections, including standard sociodemographic variables (sex, age, marital status, children, place of residence, level of education, employment), and an additional question concerning changes in economic conditions as a result of the pandemic. The variables related to the COVID-19 infection related to current or past COVID-19 infection among participants or their family members.

In a further part of the survey, 5 standardized psychometric tools were used:

#### 2.2.1. Positive and Negative Affect Schedule

In our study, a Polish adaptation (Skala Uczuć Pozytywnych i Negatywnych, SUPIN) [21] of the Positive and Negative Affect Schedule (PANAS) [22] was used. The PANAS consists of 20 items—adjectives describing positive and negative emotions. The items are rated by subjects on a 5-point scale (1 = “very slightly” or “not at all”, 2 = “a little”, 3 = “moderately”, 4 = “quite a bit”, 5 = “extremely”) in order to assess the intensity of each affect. As a result, two 10-item subscales are created that measure the positive affect (PA) and negative affect (NA). Cronbach’s α reliability indices for the scale ranged from 0.86 (PA) to 0.95 (NA).

#### 2.2.2. Mood

Mood was assessed with a Polish adaptation (Przymiotnikowa Skala Nastroju UMACL) [23] of the UWIST Mood Adjective Checklist (UMACL) [24]. The UMACL scale consists of 29 items in the form of adjectives describing mood. The surveyed people choose an answer from a 4-point scale (“definitely”, “slightly”, “slightly not”, “definitely not”), rating the applicability of each adjective to their current mood. The UMACL measures three dimensions of mood: hedonic tone (HT), tense arousal (TA) and energetic arousal (EA). The Hedonic Tone (HT) (pleasure–displeasure) scale consists of 10 items. The Tense Arousal (TA) (nervous–relaxed) scale consists of 9 items. The Energetic Arousal (EA) (energy to act) scale consists of 10 items. Cronbach’s α reliability indices for the scale ranged from 0.79 to 0.92 for the individual subscales.

#### 2.2.3. Satisfaction with Life

Satisfaction with life was assessed with a Polish adaptation (Skala Satysfakcji z Życia) [25] of the Satisfaction with Life Scale (SWLS) [26]. The SWLS contains five statements regarding one’s life. The participants are asked to rate each provided statement on a 7-point scale (1 = “strongly disagree”, 7 = “strongly agree”). Higher scores denote greater satisfaction with life. The Cronbach’s α coefficient was 0.81.

#### 2.2.4. Optimism

Optimism was measured using a Polish adaptation (Test Orientacji Życiowej) [27] of the Life Orientation Test-Revised (LOT-R) [28]. The scale consists of 10 items. The respondents are asked to rate the extent to which they agree with each item on a 5-point scale (from 0 = “strongly disagree” to 4 = “strongly agree”). The total score is calculated by adding the points from 6 diagnostic statements, ranging from 0 to 24 points, with higher scores denoting more optimism. The Cronbach’s α coefficient was 0.76.

#### 2.2.5. Coping with Stress

To measure coping with stress, the Polish adaptation (Kwestionariusz Radzenia sobie w Sytuacjach Stresowych) [29] of the Coping Inventory for Stressful Situations (CISS) [30] was used. The CISS contains 48 items describing various behaviors in stressful situations. The respondents are asked to rate the frequency of engaging in a given behavior in a stressful situation on a 5-point scale (from 1 = “never” to 5 = “very often”). The results are described in terms of three styles of coping with stress: task-oriented coping (TOC), emotion-oriented coping (EOC), and avoidance-oriented coping (AOC). The latter style may take the form of distraction (D) or social diversion (SD). The Cronbach’s α reliability indices for the scale ranged from 0.82 to 0.89 for the individual subscales.

### 2.3. Design and Procedure

Our research was an ex post-facto cross-sectional study conducted using an online survey questionnaire. The ethics approval was obtained from the Ethics Committee of the Jesuit University Ignatianum in Krakow in accordance with the principles embodied in the Declaration of Helsinki. The participants explicitly expressed their consent by checking a box after reading the instruction which explained the aims of the study, data processing, and data anonymity.

First, a survey questionnaire was developed in Google Forms, which consisted of two parts. The first one included sociodemographic variables and data related to COVID-19 infection. The second part contained standardized research tools. The study was conducted using the “snowball” method (via social media). Participation in the study was voluntary and anonymous, and the participants could resign from filling in and submitting their responses at any time. Filling in the survey took approximately 20 min.

The study was conducted from 1 December 2020 to 1 January 2021. It was a special month, because at that time, the number of new coronavirus cases and COVID-related deaths in Poland was very high (9.105 new infections and 449 deaths were recorded on 1 December 2020) [31], which resulted in tightened government restrictions. It should also be underlined that, for many Poles, December is a month of spiritual preparation for Christmas, and that restrictions limited both family contacts and active participation in religious ceremonies.

### 2.4. Data Analysis

The statistical analysis was performed with the R software, version 4.0.3 [32]. The analysis of qualitative (i.e., non-numeric) variables was performed by calculating the number and percentage of occurrences of each value. The analysis of quantitative variables (i.e., expressed in number) was performed by calculating the mean, standard deviation, median, and quartiles. The multivariate analysis of the influence of many variables on the quantitative variable was performed using the linear regression method. The results are presented as the values of the regression model parameters with a 95% confidence interval. A significance level of 0.05 was adopted in the analysis. Thus, all p values below 0.05 were interpreted as showing significant relationships.

## 3. Results

### 3.1. Participants

There were 595 respondents who participated in the research: 476 women (80.50%) and 116 men (19.50%). The respondents’ age range was from 18 to 75 years of age (M = 35.95, SD = 13.32). Table 1 shows characteristics of the sample, both in terms of the sociodemographic and COVID-related variables.

The study participants were mostly women, middle-aged, big city dwellers, with higher education, employed, and whose economic situation has not changed during pandemic. The variables related to marital status and children were evenly distributed.

#### 3.1.1. Emotions

The PANAS scale is useful for diagnosing the sign and intensity of emotions experienced by people. The scale result makes it possible to evaluate the current positive and negative emotions. The results of the PANAS are presented in Table 2.

It is assumed that people who obtain higher results in the subscale of positive affect (PA) are generally mentally healthy and socially adjusted, and they experience anxiety less frequently. In turn, people who obtain higher results in the subscale of negative affect are characterized by worse psychosocial functioning [21]. Whereas the sample was heterogenous in terms of experiencing positive emotions, more than half of the respondents displayed negative emotions in a pandemic situation. Therefore, feelings such as anxiety, fear, nervousness, and worry were common in the research group.

#### 3.1.2. Mood

In the Polish adaptation of the UMACL scale, mood is defined as “an affective experience with a moderate time of duration (at least several minutes), unrelated to an object or related to a quasi-object, which includes three dimensions of the essential affect: tense arousal, energetic arousal and hedonic tone” [23]. The results of the UMACL are presented in Table 3.

Positive mood is expressed in a high result in HT and EA, and a low result in TA. The reverse, i.e., a low level of hedonic tone, a low level of energetic arousal, and a high level of tense arousal, indicates a negative mood. The study showed a decrease in mood in the research group, as evidenced by the low level of hedonic tone (HT), which refers to pleasant-unpleasant feelings; low level of energetic arousal (EA), which refers to the energy to act; and high level of tense arousal (TA), which refers to anxiety.

#### 3.1.3. Satisfaction with Life

Apart from experiencing positive emotions and the lack of negative emotions, satisfaction with life is an element of good mood. The results of the SWLS are presented in Table 4.

No difference was observed in the number of people with low, medium, and high levels of satisfaction with life.

#### 3.1.4. Optimism

Dispositional optimism is a generalized tendency to expect good outcomes in future. Research shows that such optimism is an important predictor of a person’s wellbeing and that it facilitates success and resistance to stressful life situations, e.g., the pandemic [27]. The results of the LOT-R are presented in Table 5.

The optimistic orientation was most common among the respondents, while the pessimistic one was the second most common and the neutral one was the least frequent.

#### 3.1.5. Styles of Coping with Stressful Situations

In psychological literature, different definitions of “stress” and “coping with stress” can be found. In this research, the authors assumed that stress results from the lack of balance between demands and abilities to cope with them. Coping with stress, in turn, includes “constantly changing cognitive and behavioural efforts to manage specific external and/or internal demands that are appraised as taxing or exceeding the resources of the person” [33] (p. 141). The results obtained by the research participants in terms of their coping with stress styles are presented in Table 6.

The avoidance-oriented style was slightly dominant among the participants. Avoidance-oriented coping may take the form of distraction and social diversion. Both forms are aimed at avoiding a stressful situation [29]. Of the two forms, distraction is more often chosen. The second most frequently indicated is an emotion-oriented coping style, which includes focusing on one’s emotions and taking up actions aimed at lowering emotional tension. The least frequently chosen style is task-oriented coping (which includes taking actions aimed at solving a problem (e.g., through planning or taking up particular activities).

### 3.2. Predictors of Positive and Negative Emotions

Another issue that was analyzed was the influence of demographical variables, health situation related to COVID-19, mood, optimism, satisfaction with life, as well as styles of coping with stress, on experiencing positive and negative emotions.

The results of the analyses are presented in the Table 7.

#### 3.2.1. Predictors of Positive Emotions (PA)

The multivariate model of linear regression confirmed that significant (*p* ˂ 0.05) independent predictors of PA included: an emotion-oriented style of coping with stress (beta = −0.104; *p* = 0.001) and task-oriented coping (beta = 0.18; *p* < 0.001), level of satisfaction with life (beta = 0.235; *p* < 0.001), and level of energetic arousal in the description of mood (beta = −0.8; *p* < 0.001). A weaker predictor of experiencing positive emotions was being a man (beta = 1.935; *p* = 0.002), hedonic tone in the description of mood (beta = 0.357; *p* = 0.008), and being an employed student (beta = 2.198; *p* = 0.016). The R² coefficient for this model (PE) was 54.57%, which means that 54.57% of PA variability was explained by the variables used in the model. The remaining 45.43% depends on the variables that were not taken into account in the model and accidental factors.

#### 3.2.2. Predictors of Positive of Negative Emotions (NA)

The multivariate model of linear regression confirmed that the significant (*p* ˂ 0.05) independent predictors of NA were: tense arousal in the description of mood (beta = 1.545; *p* ˂ 0.001) and an emotion-oriented coping with stress (beta = 0.103; *p* ˂ 0.001). Other significant predictors included being over 60 years old (beta = 3.282; *p* = 0.047), living in a medium-sized city (beta = −1.606; 0.022), living in a village (beta = −1.37; *p* = 0.013), as well as decreased (beta = 1.452; *p* = 0.007) and increased (beta = 2.314; *p* = 0.007) level of life in the recent time. The R² coefficient for this model was 63.42%, which means that 63.42% of NA variability was explained by the variables used in the model. The remaining 36.58% depends on the variables that were not taken into account in the model and accidental factors.

## 4. Discussion

Experiencing negative emotions during the pandemic is a fully understandable phenomenon. In the case of 60% of respondents, the intensification of negative emotions reached a high level. Such emotions may result from a variety of factors, the importance of which may be different in various cultural contexts. In the case of the Polish respondents, this may include the following factors: the threat to one’s health and to the health of family members; isolation and, at the same time, the inability to distance oneself from people with whom we live; and economic uncertainty, together with a simultaneous crisis of trust in public institutions [8]. The objective of this research was to search for predictors of the experience of positive and negative emotions of Polish respondents during the second wave of the COVID-19 pandemic in the hope that the diagnosis of their mental condition will help design actions that might prevent negative consequences for their mental health.

Referring to the research that was conducted earlier, it is worth analyzing the key social and demographical variables. Whereas sex was not an important predictor of experiencing negative emotions, being a man was a predictor of experiencing positive emotions. In the majority of Polish analyses that were conducted earlier and that considered the sex variable, women’s results were worse as far as mental wellbeing was concerned (e.g., anxiety, stress, or depression) [6,7,9,10,17,18]. Only in one study was the difference between the sexes statistically insignificant in some measurements [13]. Women stand on the frontline in the fight against the coronavirus. In a UN report published in April 2020, a strong thesis was formulated: “The COVID-19 global crisis has made starkly visible the fact that the world’s formal economies and the maintenance of our daily lives are built on the invisible and unpaid labor of women and girls” [34]. In this situation, in which responsibility for caring for children, for the ill, and for the elderly was largely moved from the state to individuals and families, in most cases, women became the ones who had to take responsibility for it. This also limited women’s ability to work the well-paid jobs they had before the pandemic, and from a long-term perspective, it may constitute a serious obstacle on their career path [35]. Moreover, jobs performed by women are often jobs with a high risk of becoming infected with the virus (medical staff, teachers, office workers) [34]. Finally, in December, apart from the above-mentioned factors, women were burdened with preparations for Christmas which, in traditional Polish families, are mainly the responsibility of women. Thus, on the one hand, negative emotions experienced by women at that time could be based on culturally determined tasks related to unpaid and unappreciated work that involves caring for others’ needs. On the other hand, women’s negative feelings could be based on stronger social approval of experiencing such emotions by women rather than by men. However, such a trend was not confirmed by our research. Emotional costs take the form of a ricochet: a higher probability of a higher level of positive emotions among the men than among the women participating in our research.

Many analyses performed in different parts of the world have shown that the emotional distress experienced during the pandemic mainly influenced people from younger age groups. A meta-analysis of the research on emotional well-being of young people during the pandemic [36] showed that they are much more threatened with the risk of experiencing anxiety, stress, and depression than older people. In addition, young people experienced problems with sleeping [37], somatization disorders, and obsessive-compulsive disorders [38]. Stronger symptoms of emotional disorders among young people may be explained by lower psychological resilience resulting from, e.g., shorter life experience or a more drastic change in the lifestyle they led [13]. Nevertheless, in our research, the only age-related predictor of emotions was being over 60 years old, which was a predictor of negative emotions. The pandemic negatively influenced the way in which older people function because they lost the opportunity to move around, they became lonelier, and they experienced more conflicts within their families [39]. Another source of negative emotions in this age group might be older people’s increased susceptibility to contracting the virus and being more seriously affected than younger people [40]. Moreover, considering the fact that the greatest source of stress for Poles is the state of the health service [16,20], which older people use most frequently, a higher risk of experiencing negative emotions among them is perfectly understandable. Finally, for older people, isolation bears different connotations than for younger people. Older people have lower technological competences, and they often fear using new forms of media communication, which makes them feel much more isolated than young people [41].

In contrast, one of the predictors of positive emotions was combining work with studies. On the one hand, the simultaneous fulfillment of two tasks is a great challenge, especially due to the risk of losing a job to which young people working in services are often exposed, and due to dynamic changes in the system of education (the necessity to deal with the requirements of online education) [36]. On the other hand, the necessity to fulfil tasks in two social contexts at the same time increases the probability of maintaining social relationships and weakens the sense of isolation, which is one of the sources of anxiety and other disorders [42,43].

From the research, the authors have concluded that research participants living in villages and medium-sized cities experience negative emotions to a lesser degree. Living in a big city increases the risk of contracting the virus, makes it more difficult to maintain social distance, and limits the opportunity to engage in outdoor activities [19]. Furthermore, the pandemic limited people’s access to the biggest attractions connected with living in a city (access to cultural institutions and to a variety of attractive services). Other factors that increase the possibility of experiencing negative emotions may include limited and closed spaces, the necessity to maintain contact with strangers, and the sense of greater anonymity.

The strongest predictors of emotions included the styles of coping with stress. A predictor of positive emotions was a task-oriented style, while in the case of negative emotions, an emotion-oriented way of coping was a predictor. The latter style also lowered the opportunity to experience positive emotions. Task-oriented coping relates to an important element of constructive coping with pandemic stress: control over one’s surrounding reality [44]. This style involves reformulating the evaluation of the situation from a threat to a challenge or a task to be fulfilled. In the context of the pandemic, the style may be reflected in behaviors that reduce the risk of becoming infected with the virus, as well as actions such as planning everyday routines, looking for reliable information about the virus, etc.

In our research, emotion-oriented coping was a predictor of negative emotions and the original affect dimension related to lower mood (tense arousal). The adaptive way of dealing with negative emotions involves recognizing, naming, and accepting emotions that accompany difficult situations. The emotion-oriented style of coping with stress, the essence of which is focusing on one’s own emotions and taking up actions aimed at reducing emotional tension, seems to be a non-adaptive solution, especially because, in the case of uncertain and uncontrollable conditions, these actions are doomed to failure. Continuous tense arousal and energetic arousal related to our body’s preparation to respond to threats results in exhaustion. However, an important predictor of positive emotions was hedonic tone.

Similar to other analyses [45,46], our research has confirmed the relationship between mental wellbeing and satisfaction with life. Comparing this conclusion with the statement that an increased or decreased standard of living within the last 10 months is an important predictor of negative emotions, it could be noticed that one of the protective factors is the opportunity to use the resources gathered during the pandemic and to maintain a sense of stability/unchangeability in a changing world.

## 5. Conclusions

Surveys that diagnose the mental state of people in different countries are very useful in preventing negative consequences for their inhabitants’ mental health. Such a diagnosis should take into account the high dynamic of changes people face during a pandemic, as well as different ways of experiencing and interpreting pandemic stress by people from a variety of cultural contexts. In the Polish reality, people are not worried about their own illness or death to a high degree, but they are concerned about their family members’ health and about the crisis of trust in governmental institutions during the pandemic.

In the presented research, which was conducted during the second wave of the pandemic (December 2020), 60% of the participants revealed a high level of negative emotions. Due to the possible connection between the high intensity of negative emotions and negative consequences for one’s mental health, it is important for researchers to look for factors that increase the risk of experiencing negative feelings. Significant predictors of negative emotions include mood-related tense arousal in the description of mood and an emotion-oriented style of coping with stress. On the other hand, important predictors of positive emotions are a task-oriented style of coping with stress, level of satisfaction with life, and hedonic tone in the description of mood. These aspects may become the basic indicators for specialists who will work on preventive actions and psychological care.

In addition, it is worth focusing on supporting particular at-risk groups, i.e., people over 60 years old (e.g., through increasing their online activities) or women (through increasing their chances to experience good emotions by appreciating the value of their unpaid work).

It should be emphasized that these results must be considered in the light of numerous limitations. Adults of different ages (i.e., over 18 years old) were recruited for the research. However, because of our recruitment method (i.e., snowball sampling), both men, people with primary/middle school and vocational education, and older people were underrepresented in the research sample. Another limitation is related to the type of the research. A better solution would be longitudinal research, which would allow to make reliable conclusions about the change and its dynamics in the psychological wellbeing of the sample. Finally, the research was conducted mainly by means of the Internet to provide comfort and safety to the participants. As a result, the sample consisted primarily of people who have access to the Internet. The abovementioned limitations make it impossible to generalize the research results as representative of the population as a whole.

Despite the limitations, the research results obtained shed some light on the emotional wellbeing of adult Poles during the second wave of the COVID-19 pandemic. These findings are important because the intensification of negative emotions can contribute to problems not only in mental health, but also in everyday activities, such as study, work, social relations, or sexual contacts. The authors are aware that further research should be conducted to increase the number of participants in each age group, from children to seniors.

In the context of an unpredictable future, researchers face the task of monitoring the emotional condition of the general population and of particular at-risk groups in order to inspire practical preventive and therapeutic actions, as well as social initiatives that reinforce solidarity, mutual care, and responsibility.

## Figures and Tables

**Table 1:** Characteristics of the sample: sociodemographic and COVID-19-related variables.

Sociodemographic and COVID-Related Variables		*n*	%
Sex	Female	476	80.50
	Male	116	19.50
Age	Under 22	124	20.84
	23–34 years of age	156	26.22
	35–60 years of age	280	47.06
	Over 60 years of age	35	5.88
Marital status	Single	259	43.52
	Married	297	49.92
	Others	39	6.56
Children	No	285	47.90
	Yes	310	52.10
Place of residence	Big city	277	46.55
	Medium-sized city	86	14.45
	Small city	62	10.42
	Village	170	28.57
Education	Higher	353	59.33
	Secondary	64	10.76
	Other	178	29.92
Employment	Student	114	19.16
	Employed	320	53.78
	Not employed	63	10.59
	Employed student	98	16.47
Economic conditions	Not changed	357	60.00
	Decreased	185	31.09
	Improved	53	8.91
Have you had COVID 19?	No	462	77.65
	Yes	133	22.35
Has anyone in your family had COVID 19?	No	306	51.43
	Yes	289	48.57

**Table 2:** The characteristics of the study participants regarding the level of positive and negative affect.

Level	PANAS	
	PA ^1^	NA ^2^
Low	227 (38.15%)	65 (10.92%)
Medium	180 (30.25%)	173 (29.08%)
High	188 (31.60%)	357 (60.00%)

^1^ PA—Positive Affect, ^2^ NA—Negative Affect.

**Table 3:** The characteristics of the study participants regarding the level of mood dimensions.

Level	UMACL		
	HT ^1^	TA ^2^	EA ^3^
Low	558 (93.78%)	11 (1.85%)	548 (92.10%)
Medium	37 (6.22%)	143 (24.03%)	40 (6.72%)
High	0 (0.00%)	441 (74.12%)	7 (1.18%)

^1^ HT—Hedonic Tone, ^2^ TA—Tense Arousal, ^3^ EA—Energetic Arousal.

**Table 4:** The characteristics of the study participants regarding the level of satisfaction with life.

SWLS	
Level	
Low	194 (32.61%)
Medium	186 (31.26%)
High	215 (36.13%)

**Table 5:** The characteristics of the study participants regarding life orientation.

Life Orientation	LOT-R
Pessimistic	195 (32.77%)
Neutral	171 (28.74%)
Optimistic	229 (38.49%)

**Table 6:** The characteristics of the study participants regarding styles of coping with stress.

Level	CISS				
	TOC ^1^	EOC ^2^	AOC ^3^	D ^4^	SD ^5^
Low	174 (29.24%)	139 (23.36%)	119 (20.00%)	89 (14.96%)	165 (27.73%)
Medium	226 (37.98%)	233 (39.16%)	241 (40.50%)	284 (47.73%)	236 (39.66%)
High	195 (32.77%)	223 (37.48%)	235 (39.50%)	222 (37.31%)	194 (32.61%)

^1^ TOC—task-oriented coping, ^2^ EOC—emotion-oriented coping, ^3^ AOC—avoidance-oriented coping, ^4^ D—distraction, ^5^ SD—social diversion.

**Table 7:** Predictors of positive and negative emotions—linear regression results.

Variable		PA ^1^				NA ^2^			
		Parameter	95% CI		*p*	Parameter	95% CI		*p*
Sex	Female	ref.				ref.			
	Male	1.935	0.692	3.179	0.002 *	−0.234	−1.407	0.939	0.696
Age	Up to 22	ref.				ref.			
	23–34	0.666	−1.174	2.506	0.479	1.063	−0.673	2.799	0.231
	35–60	−0.951	−3.565	1.663	0.476	1.189	−1.277	3.655	0.345
	Over 60	−1.265	−4.688	2.159	0.469	3.282	0.052	6.512	0.047 *
Marital status	Single/in informal relationships	ref.				ref.			
	Married	−0.566	−2.237	1.105	0.507	0.557	−1.019	2.134	0.489
	Other	1.231	−1.157	3.618	0.313	0.738	−1.514	2.99	0.521
Children	No	ref.				ref.			
	Yes	0.18	−1.536	1.896	0.837	−0.468	−2.087	1.151	0.571
Place of residence	Big city	ref.				ref.			
	Medium-sized city	−0.898	−2.35	0.553	0.226	−1.606	−2.975	−0.237	0.022 *
	Small city	1.264	−0.367	2.895	0.129	0.375	−1.164	1.913	0.633
	Village	0.087	−1.059	1.234	0.882	−1.37	−2.452	−0.289	0.013 *
Education	Higher education	ref.				ref.			
	Secondary education	−1.019	−2.666	0.628	0.226	−0.009	−1.563	1.545	0.991
	Other	0.41	−1.12	1.939	0.6	−0.728	−2.171	0.715	0.323
Employment	Student	ref.				ref.			
	Employed	1.852	−0.456	4.16	0.116	−0.117	−2.294	2.061	0.916
	Not employed	1.74	−0.922	4.401	0.201	−0.088	−2.599	2.422	0.945
	Employed student	2.198	0.419	3.977	0.016 *	0.698	−0.98	2.376	0.415
Economic conditions	Not changed	ref.				ref.			
	Decreased	−0.096	−1.202	1.009	0.864	1.452	0.409	2.495	0.007 *
	Improved	−0.787	−2.549	0.975	0.382	2.314	0.652	3.976	0.007 *
Have you had COVID 19?	No	ref.				ref.			
	Yes	−0.881	−2.106	0.345	0.16	0.407	−0.749	1.564	0.49
Has anyone in your family had COVID 19?	No	ref.				ref.			
	Yes	−0.243	−1.259	0.773	0.64	−0.2	−1.159	0.758	0.682
UMACL: HT ^3^		0.357	0.096	0.618	0.008 *	0.028	−0.218	0.275	0.821
UMACL: TA ^4^		−0.144	−0.331	0.042	0.13	1.545	1.369	1.721	<0.001 *
UMACL: EA ^5^		−0.8	−0.965	−0.634	<0.001 *	0.138	−0.018	0.294	0.083
SWLS		0.235	0.131	0.338	<0.001 *	−0.038	−0.136	0.06	0.442
LOT-R		−0.019	−0.152	0.113	0.774	−0.089	−0.214	0.036	0.165
CISS: TOC ^6^		0.18	0.114	0.247	<0.001 *	0.032	−0.031	0.096	0.313
CISS: EOC ^7^		−0.104	−0.165	−0.043	0.001 *	0.103	0.045	0.161	<0.001 *
CISS: AOC ^8^		0.009	−0.293	0.312	0.952	−0.163	−0.448	0.123	0.264
CISS: D ^9^		0.013	−0.34	0.365	0.944	0.213	−0.119	0.545	0.21
CISS: SD ^10^		0.154	−0.234	0.541	0.437	0.156	−0.21	0.521	0.405

* *p* —values below 0.05; ^1^ PA—Positive Affect, ^2^ NA—Negative Affect, ^3^ HT—Hedonic Tone, ^4^ TA—Tense Arousal, ^5^ EA—Energetic Arousal, ^6^ TOC—task-oriented coping, ^7^ EOC—emotion-oriented coping, ^8^ AOC—avoidance-oriented coping, ^9^ D—distraction, ^10^ SD—social diversion.

## Data Availability

The data presented in this study are available on request from the corresponding author.
